# Supplementary material for: Causal Relationship between Aging and Anorexia Nervosa: A White-Matter-Microstructure-Mediated Mendelian Randomization Analysis
Source: Biomedicines. 2024 Aug 16;12(8):1874. doi: 10.3390/biomedicines12081874 (PMC11351342; doi:10.3390/biomedicines12081874)
Supplement: Supplementary file 1 [file biomedicines-12-01874-s001.zip › Supplementary Figure/Figure S4/Singe SNP plot of MR analysis for TL to ubm-b-2115.pdf]

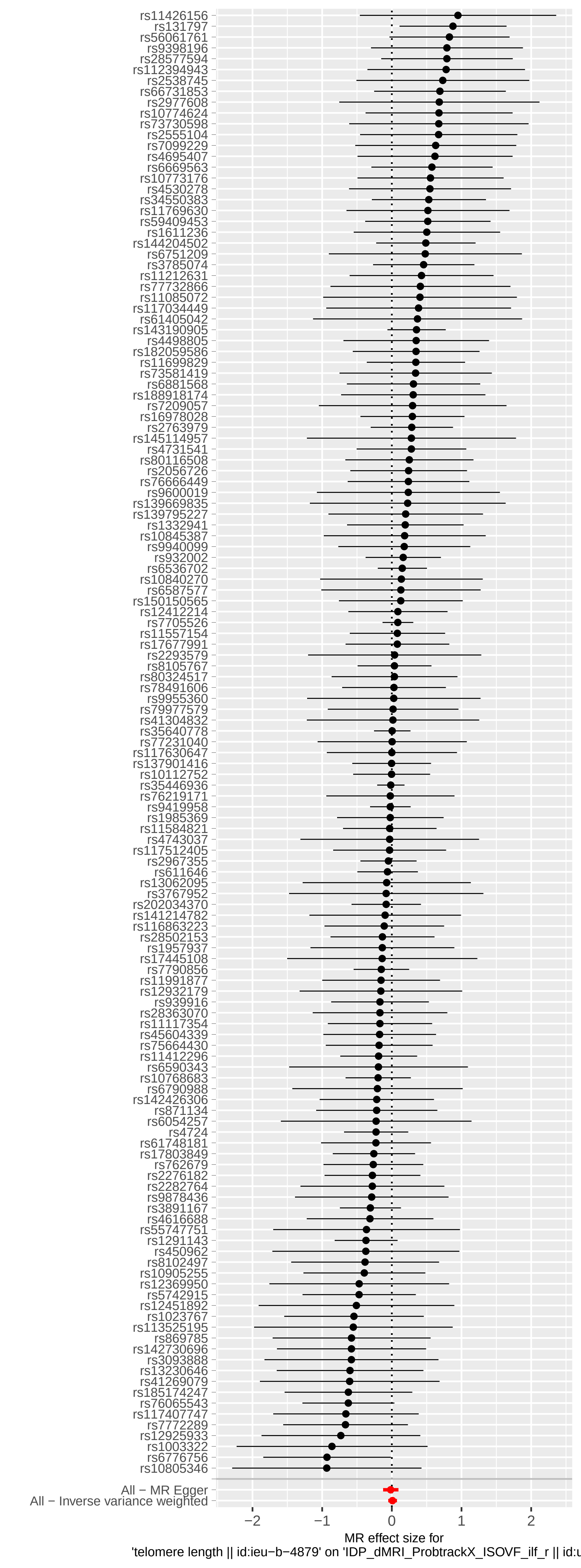
rs11426156
rs131797
rs56061761
rs9398196
rs28577594
rs112394943
rs2538745
rs66731853
rs2977608
rs10774624
rs73730598
rs2555104
rs7099229
rs4695407
rs6669563
rs10773176
rs4530278
rs34550383
rs11769630
rs59409453
rs1611236
rs144204502
rs6751209
rs3785074
rs11212631
rs77732866
rs11085072
rs117034449
rs61405042
rs143190905
rs4498805
rs182059586
rs11699829
rs73581419
rs6881568
rs188918174
rs7209057
rs16978028
rs2763979
rs145114957
rs4731541
rs80116508
rs2056726
rs76666449
rs9600019
rs139669835
rs139795227
rs1332941
rs10845387
rs9940099
rs932002
rs6536702
rs10840270
rs6587577
rs150150565
rs12412214
rs7705526
rs11557154
rs17677991
rs2293579
rs8105767
rs80324517
rs78491606
rs9955360
rs79977579
rs41304832
rs35640778
rs77231040
rs117630647
rs137901416
rs10112752
rs35446936
rs76219171
rs9419958
rs1985369
rs11584821
rs4743037
rs117512405
rs2967355
rs611646
rs13062095
rs3767952
rs202034370
rs141214782
rs116863223
rs28502153
rs1957937
rs17445108
rs7790856
rs11991877
rs12932179
rs939916
rs28363070
rs11117354
rs45604339
rs75664430
rs11412296
rs6590343
rs10768683
rs6790988
rs142426306
rs871134
rs6054257
rs4724
rs61748181
rs17803849
rs762679
rs2276182
rs2282764
rs9878436
rs3891167
rs4616688
rs55747751
rs1291143
rs450962
rs8102497
rs10905255
rs12369950
rs5742915
rs12451892
rs1023767
rs113525195
rs869785
rs142730696
rs3093888
rs13230646
rs41269079
rs185174247
rs76065543
rs117407747
rs7772289
rs12925933
rs1003322
rs6776756
rs10805346
All − MR Egger
All − Inverse variance weighted
−2
−1
0
1
2
MR effect size for
'telomere length || id:ieu−b−4879' on 'IDP_dMRI_ProbtrackX_ISOVF_ilf_r || id:u
